# miR-889-3p targets SLC35E1 in leprosy: Implications for therapeutic monitoring

**DOI:** 10.1371/journal.pntd.0014653

**Published:** 2026-08-25

**Authors:** Yang Li, Lei Chen, Zepu Guan, Liting Cai, Buning Ye, Xiaohua Wang

**Affiliations:** Department of Dermatology, Dermatology Hospital, Southern Medical University, Guangzhou, Guangdong, China; University of Bremen: Universitat Bremen, GERMANY

## Abstract

**Background:**

Leprosy is a chronic infectious disease caused by Mycobacterium leprae (M. leprae). Despite significant progress in disease control, the molecular mechanisms regulating host responses to M. leprae infection remain incompletely understood. MicroRNAs (miRNAs) are important post-transcriptional regulators that modulate immune responses and host–pathogen interactions in many infectious diseases. However, the specific miRNA-mediated regulatory networks involved in leprosy and their potential implications for monitoring therapeutic responses remain poorly defined.

**Methods:**

Skin lesions, serum, and nasal mucosa samples were collected from patients with multibacillary (MB) and paucibacillary (PB) leprosy, as well as from healthy controls. Differentially expressed miRNAs were identified using an Agilent Human miRNA Microarray and validated by quantitative reverse transcription polymerase chain reaction (qRT-PCR). The interaction between miR-889-3p and its predicted target gene SLC35E1 was examined using dual-luciferase reporter assays. Protein expression of SLC35E1 in skin tissues was evaluated by immunohistochemistry (IHC), and its association with the bacterial index (BI) was analyzed.

**Results:**

Microarray analysis identified multiple miRNAs with differential expression between leprosy patients and healthy controls across skin lesions, serum, and nasal mucosa samples. Among these candidates, miR-889-3p was consistently upregulated and showed higher expression particularly in multibacillary (MB) patients. qRT-PCR validation confirmed elevated miR-889-3p expression in the serum, skin, and nasal mucosa of MB patients compared with healthy controls, whereas no significant difference was observed in paucibacillary (PB) patients. Notably, miR-889-3p levels decreased significantly after multidrug therapy (MDT) and approached levels observed in healthy controls. Dual-luciferase reporter assays demonstrated that miR-889-3p directly interacts with the 3′ untranslated region (3′UTR) of SLC35E1 and significantly increased reporter activity in the wild-type construct, whereas no effect was observed in the mutant construct, supporting a sequence-specific regulatory interaction.Immunohistochemical analysis further revealed increased SLC35E1 expression in leprosy lesions and a strong positive correlation between SLC35E1 expression and the bacteriological index (r = 0.926, p < 0.001).

**Conclusion:**

These findings demonstrate that miR-889-3p targets SLC35E1 in leprosy and is associated with bacterial burden and treatment-related changes following multidrug therapy. The dynamic alteration of miR-889-3p expression during therapy suggests that this miRNA may serve as a potential molecular biomarker for monitoring therapeutic response in leprosy. In addition, the identification of the miR-889-3p/SLC35E1 regulatory interaction provides new insights into miRNA-mediated regulatory mechanisms involved in host responses to Mycobacterium leprae infection.

## Author summary

Leprosy is a chronic infectious disease caused by Mycobacterium leprae, which remains a public health concern in some regions despite effective multidrug therapy. Although treatment can successfully eliminate infection, the biological processes involved in host responses to M. leprae infection and the identification of markers that reflect disease status remain incompletely understood. In this study, we investigated small regulatory molecules called microRNAs, which can control gene activity and influence immune responses during infection. By analyzing samples from patients with leprosy and healthy individuals, we identified miR-889-3p as a molecule that was increased in patients with a higher bacterial burden and decreased after multidrug therapy. We further found that miR-889-3p interacts with SLC35E1, a potential target gene, and that SLC35E1 expression in skin lesions was associated with the bacterial index of patients. These findings provide new insights into how microRNAs may participate in the host response to M. leprae infection. Although further studies with larger patient groups are needed, miR-889-3p and SLC35E1 may represent promising candidates for understanding disease activity and treatment-related changes in leprosy.

## Introduction

Leprosy, caused by Mycobacterium leprae, is a chronic infectious disease that primarily affects the skin and peripheral nerves [1,2]. Although the global incidence of leprosy has declined due to widespread multidrug therapy (MDT), the disease remains endemic in several countries, including India, Brazil, and Indonesia [3,4]. The clinical manifestations of leprosy reflect a spectrum of host immune responses to M. leprae infection [1,5]. For clinical management and treatment, patients are operationally classified by the World Health Organization (WHO) into paucibacillary (PB) and multibacillary (MB) leprosy according to bacterial burden. MB disease is characterized by a higher bacillary load and impaired cell-mediated immunity, whereas PB disease is associated with a lower bacillary burden and stronger cellular immune responses. [1,5,6]. Despite advances in treatment, reliable molecular indicators that reflect disease activity and therapeutic response remain limited [2,7].

MicroRNAs (miRNAs) are small non-coding RNAs that regulate gene expression at the post-transcriptional level by binding to complementary sequences within target mRNAs [8]. Increasing evidence indicates that miRNAs play important roles in immune regulation and host–pathogen interactions [8–10]. Dysregulated miRNA expression has been reported in several infectious and autoimmune diseases, including tuberculosis, systemic lupus erythematosus, and rheumatoid arthritis [9,11,12]. In the context of leprosy, M. leprae infection has been shown to modulate host miRNA expression patterns [13,14]. For example, M. leprae–induced miR-21 suppresses the vitamin D–dependent antimicrobial pathway and facilitates bacterial survival [15]. Other studies have identified distinct miRNA expression signatures in leprosy skin lesions and peripheral blood that may distinguish between clinical forms of the disease [13,14]. More recently, circulating miRNAs such as hsa-miR-144-5p and miR-20a-5p have been proposed as minimally invasive biomarkers for early diagnosis and disease stratification [16,17]. These findings suggest that miRNAs may contribute to the regulation of host immune responses during M. leprae infection and may also serve as potential molecular biomarkers [8,16]. However, the miRNA-mediated regulatory mechanisms involved in leprosy pathogenesis remain incompletely understood, and the potential value of specific miRNAs for monitoring disease activity and treatment response has not been fully elucidated.

In the present study, we performed miRNA expression profiling in multiple biological samples from leprosy patients and healthy controls to identify differentially expressed miRNAs. Among the candidates identified, miR-889-3p was selected for further investigation due to its consistent differential expression across multiple sample types. We further examined its interaction with the predicted target gene SLC35E1 and evaluated its association with bacterial burden and treatment status. Our findings provide new insights into the miRNA-mediated regulatory network in leprosy and suggest that the miR-889-3p/SLC35E1 axis may have potential implications for monitoring therapeutic response.Materials and Methods

## Materials and methods

### Ethics statement

All participants provided written informed consent. The study was approved by the Institutional Ethics Committee of Southern Medical University (Approval No. 2023184).

This study was designed as an exploratory observational investigation. Patients with newly diagnosed leprosy were recruited from the Department of Dermatology, Southern Medical University, Guangzhou, China. The diagnosis of leprosy was established based on clinical manifestations, histopathological examination, bacterial microscopy, and qPCR confirmation.Patients were classified as paucibacillary (PB) or multibacillary (MB) according to the World Health Organization (WHO) operational classification based on clinical findings (including the number of skin lesions) together with bacteriological examination (slit-skin smear microscopy).. Healthy individuals without a history of leprosy or other chronic inflammatory diseases were recruited as controls. Because this investigation was designed as an exploratory study, no formal sample size calculation was performed. The sample size was determined according to the availability of clinically confirmed cases during the study period.

### Sample collection and study cohorts

The study consisted of three experimental stages: microarray screening, qRT-PCR validation, and immunohistochemical analysis. Peripheral blood, skin biopsy, and nasal mucosa samples were immediately preserved in RNA stabilization reagent after collection and stored at −80°C until RNA extraction. Total RNA was extracted within one month after collection. RNA purity was assessed using a NanoDrop spectrophotometer, and only samples with an A260/A280 ratio between 1.8 and 2.0 were included for downstream analyses. RNA integrity was not assessed using a Bioanalyzer.

#### Microarray screening cohort.

For the initial miRNA screening stage, skin lesion biopsies, serum samples, and nasal mucosa swabs were collected from eight leprosy patients (4 PB and 4 MB) and three healthy controls.

#### qRT-PCR validation cohort.

For validation analysis, peripheral blood samples were collected from 21 newly diagnosed leprosy patients (7 PB and 14 MB) and three healthy controls. For leprosy patients, blood samples were obtained at two time points: before initiation of multidrug therapy (MDT), six months after completion of MDT, These paired samples were obtained from the same individuals to evaluate changes in miRNA expression during treatment. Patients received standard World Health Organization–recommended multidrug therapy (MDT): PB patients: rifampicin + dapsone for 6 months. MB patients: rifampicin + dapsone + clofazimine for 12 months

Patients receiving systemic corticosteroids for type 1 or type 2 leprosy reactions, other immunosuppressive medications, or presenting with significant concurrent systemic illnesses were predefined exclusion criteria. The validation cohort included both newly diagnosed and follow-up patients. Follow-up patients were enrolled only if they had remained clinically stable before enrollment, without clinical evidence of leprosy reactions or treatment with systemic corticosteroids during this period. No otherwise eligible patients met the predefined exclusion criteria; therefore, no participants were excluded after screening.

#### Immunohistochemistry.

For immunohistochemical analysis, skin biopsy specimens were collected from 16 leprosy patients, including 12 MB and 4 PB cases. Importantly, paired biopsy samples were obtained from the same patients both before treatment and after completion of MDT, allowing evaluation of changes in SLC35E1 protein expression during therapy. In addition, 10 normal skin samples from healthy individuals were used as controls. Paraffin-embedded sections were stained using an anti-SLC35E1 antibody. Protein expression in the epidermis and dermis was evaluated using the H-score method, which combines staining intensity and the percentage of positive cells. All slides were independently assessed by two pathologists blinded to the clinical information. In addition, the relationship between SLC35E1 expression and bacterial load was evaluated. The bacteriological index (BI) for each patient was determined using slit-skin smear microscopy according to the Ridley logarithmic scale. The correlation between SLC35E1 expression levels, quantified by H-score, and BI values was subsequently assessed.

### miRNA microarray analysis

Total RNA was extracted using TRIzol reagent according to the manufacturer’s instructions. RNA quantity and integrity were evaluated using a NanoDrop spectrophotometer and the Agilent 2100 Bioanalyzer. For the subsequent qRT-PCR validation, RNA was extracted using a commercial RNA isolation kit according to the manufacturer’s instructions. Although different extraction methods were used for the two experimental platforms, RNA quality and purity were evaluated before downstream analyses, and only samples meeting the predefined quality criteria were included.miRNA expression profiling was performed using the Agilent Human miRNA Microarray platform. Data normalization and differential expression analysis were conducted using GeneSpring GX software. miRNAs with |log_2_ fold change| ≥ 1 and P < 0.05 were considered differentially expressed (S1 Table).

### qRT-PCR validation

Total RNA was isolated from serum, skin, and nasal mucosa samples using a commercial RNA extraction kit following the manufacturer’s instructions. Reverse transcription was performed using a miRNA-specific reverse transcription kit to generate complementary DNA (cDNA). Quantitative real-time PCR (qRT-PCR) was performed using RiboBio-specific primers and SYBR Green chemistry on a real-time PCR detection system. The small nuclear RNA U6 was used as an endogenous reference gene for normalization. Relative miRNA expression levels were calculated using the 2^-ΔΔCt method. Primer sequences were adopted from previously published studies or designed according to the mature miRNA sequences available in miRBase and are listed in S1 Table. PCR amplification conditions were: 95 °C for 10 min, followed by 40 cycles of, 95 °C for 15 s, 60 °C for 60 s. All reactions were performed in triplicate.Quantitative real-time PCR was performed using a Bio-Rad Real-Time PCR Detection System (Bio-Rad Laboratories, Hercules, CA, USA). Following amplification, melting-curve analysis was performed to verify the specificity of the PCR products. Only reactions exhibiting a single specific melting peak were included in the final analysis.

### Target prediction and dual-luciferase reporter assay

Potential target genes of candidate miRNAs were predicted using TargetScan and miRDB databases. Only genes predicted by both databases were retained to increase prediction reliability. The 3′ untranslated region (3′UTR) of SLC35E1, including wild-type and mutant sequences, was cloned into luciferase reporter plasmids. HEK293T cells were co-transfected with the reporter constructs together with miR-889-3p mimics or control sequences. Luciferase activity was measured using the Dual-Luciferase Reporter Assay System according to the manufacturer’s instructions.

### Bioinformatic and functional enrichment analysis

Predicted target genes were subjected to Gene Ontology (GO) and Kyoto Encyclopedia of Genes and Genomes (KEGG) enrichment analysis using the DAVID platform. Only enrichment results with P < 0.05 were considered statistically significant.

### Statistical analysis

Statistical analyses were performed using GraphPad Prism 9. Data are presented as mean ± standard deviation (SD). For qRT-PCR validation, comparisons between independent groups were analyzed using non-parametric tests because of the small sample size. Pre- and post-MDT qRT-PCR results were interpreted based on the available summarized expression data; retrospective paired analyses could not be performed because the original individual-level Ct values were not available. For immunohistochemical H-score comparisons, Student’s t-test was used where appropriate. Fisher’s exact test was used for categorical comparisons of SLC35E1 expression levels. Pearson correlation analysis was used to evaluate the relationship between SLC35E1 H-score and the bacteriological index (BI). A P value < 0.05 was considered statistically significant.

### Data availability

Datasets related to this article can be found at: yang, li (2026), “miR-889-3p Targets SLC35E1 in Leprosy: Implications for Therapeutic Monitoring”, Mendeley Data, V1, https://doi.org/10.17632/x2jmhnxj99.1 [https://data.mendeley.com/datasets/x2jmhnxj99/1], hosted at [Mendeley Data]

The dataset deposited in Mendeley Data contains RNA quality control information (including RNA concentration, OD260/280 ratio, OD260/230 ratio, sample volume, and RNA quantity) generated prior to downstream analyses. The original individual-level qRT-PCR Ct values used to generate the summarized expression data are no longer available.

## Results

### Sample characteristics

**Fig 1:**
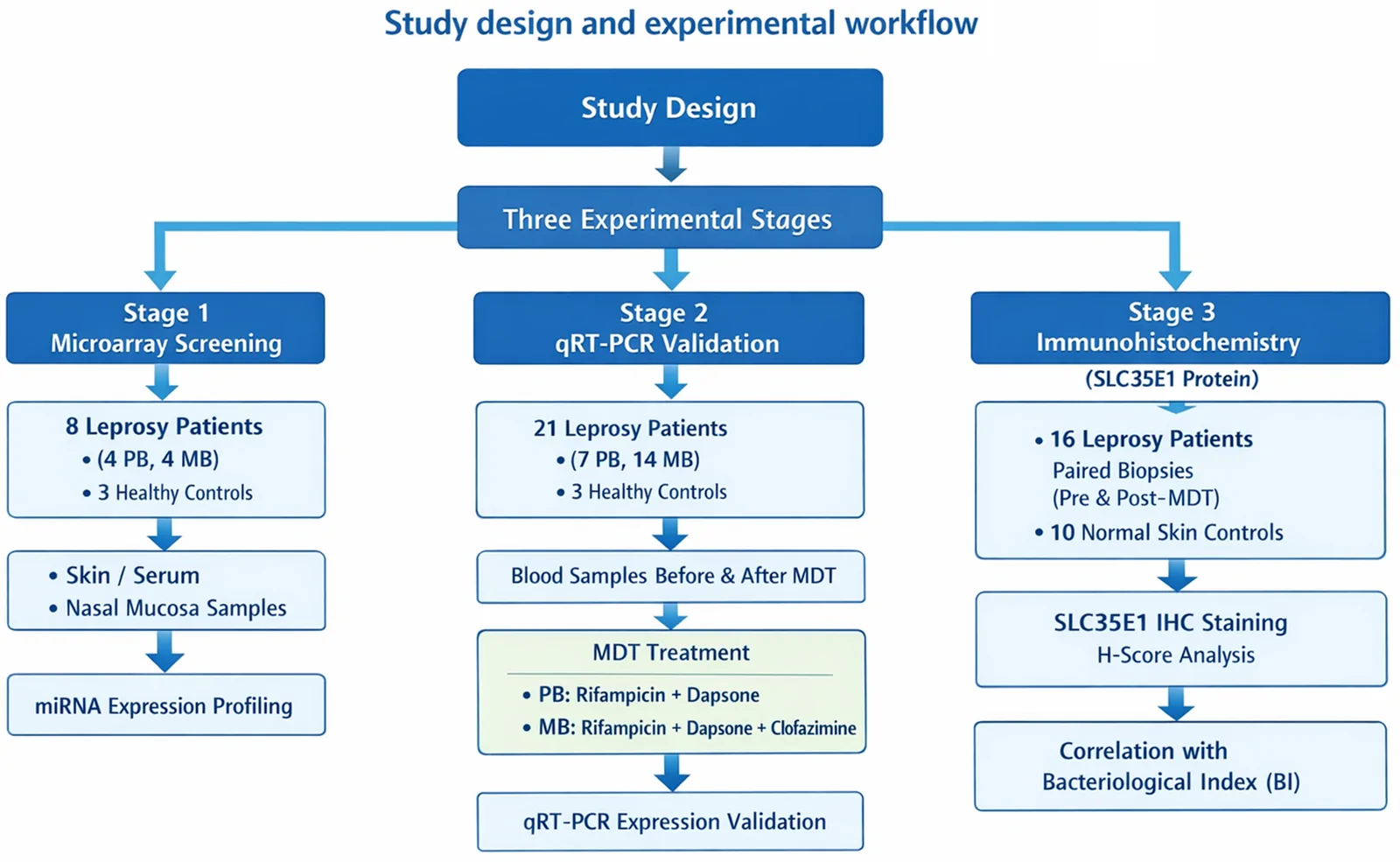
Study design and experimental workflow. The study comprised three experimental stages: microarray screening of miRNA expression profiles in skin, serum, and nasal mucosa samples; qRT-PCR validation of miRNA expression before and after multidrug therapy (MDT); and immunohistochemical evaluation of SLC35E1 expression in paired skin biopsies before and after MDT, followed by analysis of its correlation with the bacteriological index (BI). PB, paucibacillary; MB, multibacillary; qRT-PCR, quantitative reverse transcription polymerase chain reaction; MDT, multidrug therapy; IHC, immunohistochemistry; BI, bacteriological index.

A total of 21 newly diagnosed leprosy patients and three healthy controls were included in the validation cohort. According to the WHO operational classification, patients were categorized into PB (n = 7) and MB (n = 14) groups.. Among the 21 patients, 12 were male (57.2%) and 9 were female (42.8%). At diagnosis, 15 patients (71.4%) presented with more than five skin lesions, while six patients (28.6%) had five or fewer lesions. Peripheral nerve involvement was observed in 12 patients (57.1%), and three patients (14.3%) presented with grade 2 disability. For the microarray screening stage, skin lesion biopsies, serum samples, and nasal mucosa swabs were collected from eight leprosy patients (4 PB and 4 MB) and three healthy controls. A schematic overview of sample allocation across the discovery and validation stages is shown in Fig 1.

### miRNA expression profiling

**Fig 2:**
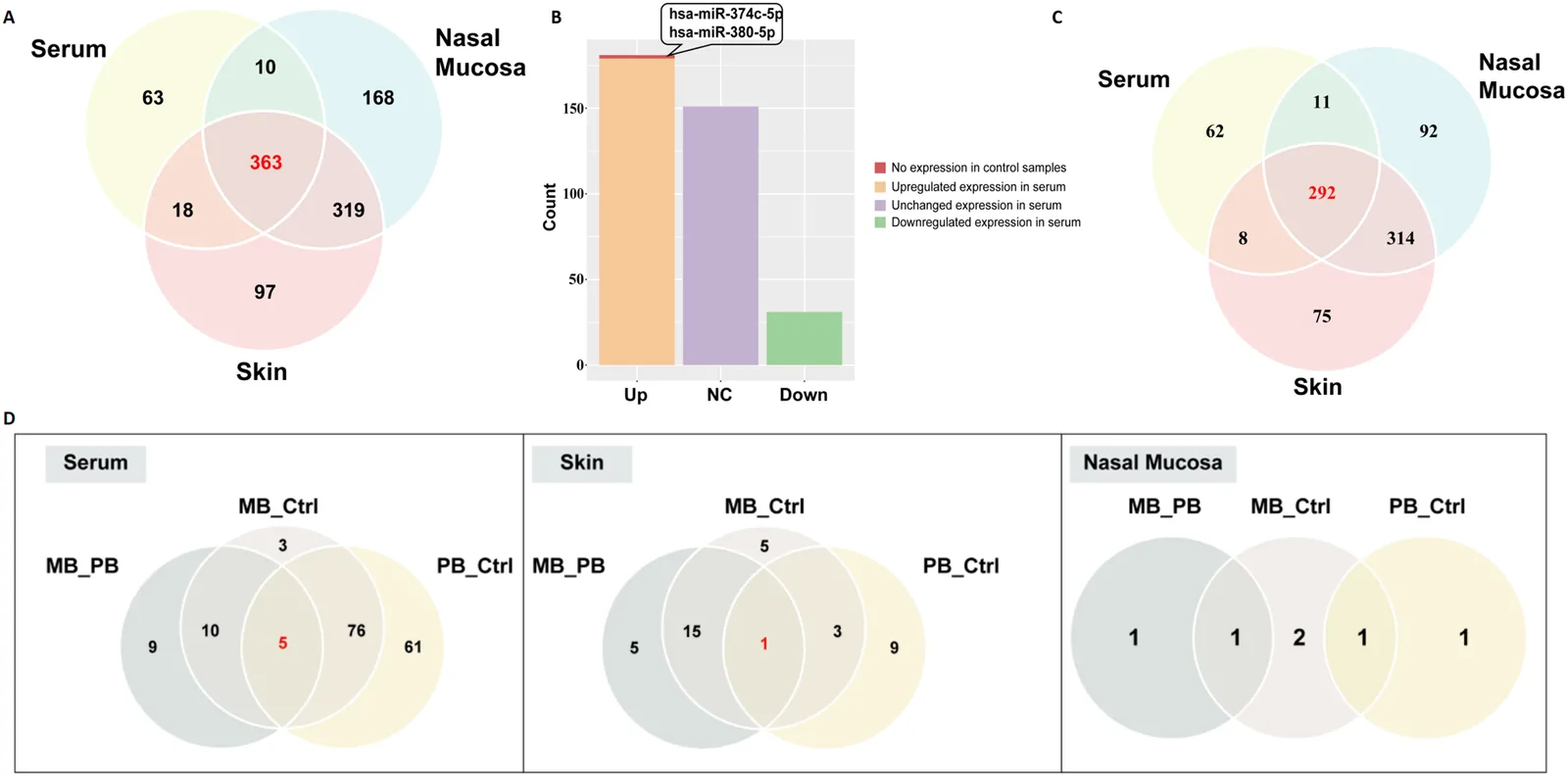
Overview of miRNA expression profiling and sample allocation. (A) Venn diagram showing overlapping miRNA expression among skin lesion, serum, and nasal mucosa samples in paucibacillary (PB) patients. (B) Distribution of miRNAs detected in serum samples of PB patients. (C) Venn diagram showing overlapping miRNA expression across the three sample types in multibacillary (MB) patients. (D) Venn diagram showing differentially upregulated miRNAs identified in MB.samples.

**Fig 3:**
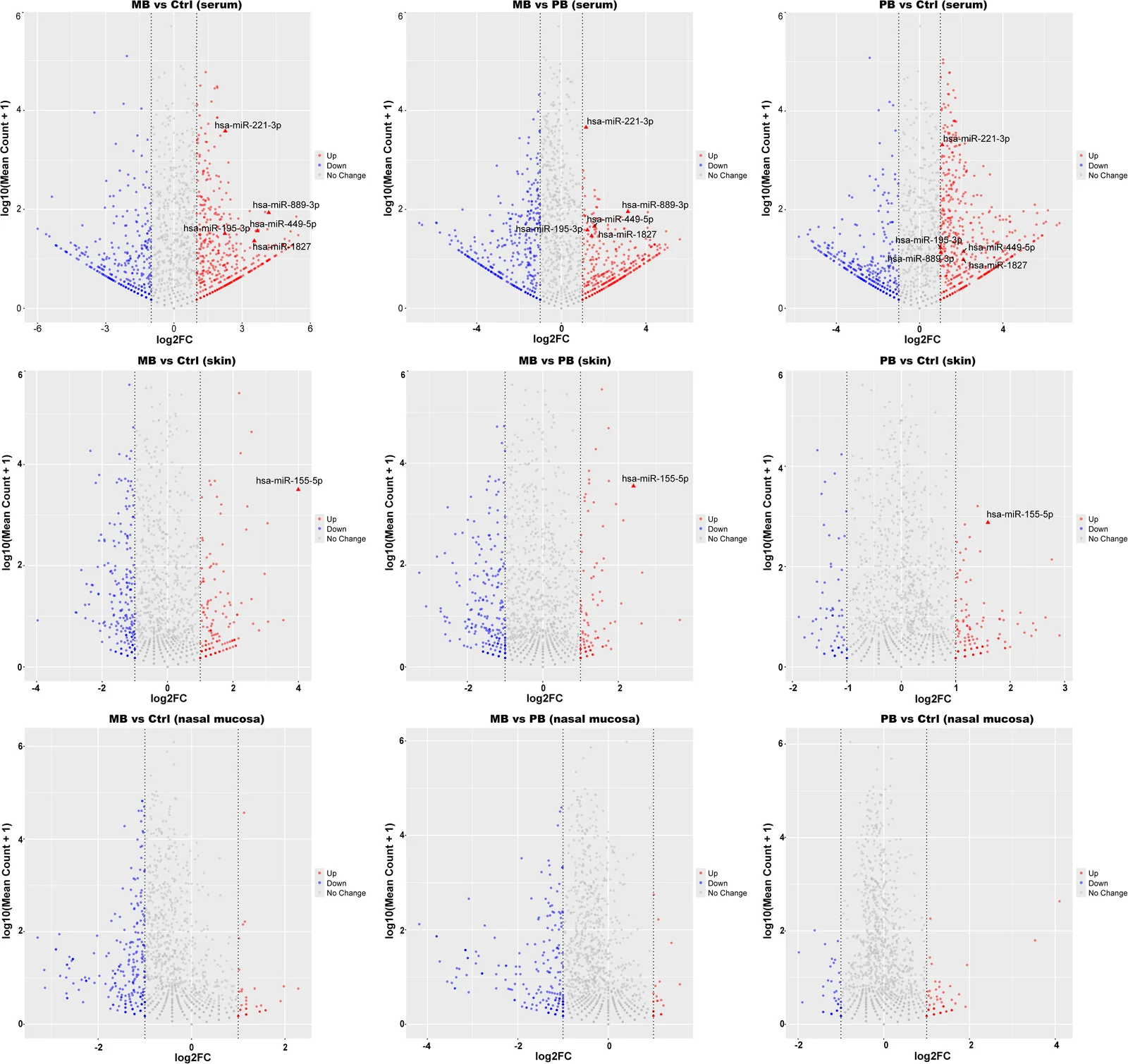
Identification of differentially expressed miRNAs in leprosy patients. Volcano plots illustrating differential miRNA expression between leprosy patients and healthy controls across three biological sample types: serum, skin lesions, and nasal mucosa. Red triangles indicate miRNAs that were consistently detected across all three sample types and showed significant differential expression in both PB and MB groups compared with healthy controls.

Microarray screening identified multiple miRNAs showing differential expression between leprosy patients and healthy controls across serum, skin lesions, and nasal mucosa samples. Among these candidates, miR-889-3p emerged as one of the most consistently upregulated miRNAs, particularly in multibacillary (MB) patients. Venn diagram analysis demonstrated overlapping miRNA expression patterns across the three biological compartments, and several miRNAs showed consistent dysregulation in MB samples (Figs 2 and 3).

### qRT-PCR validation of differentially expressed miRNAs

**Fig 4:**
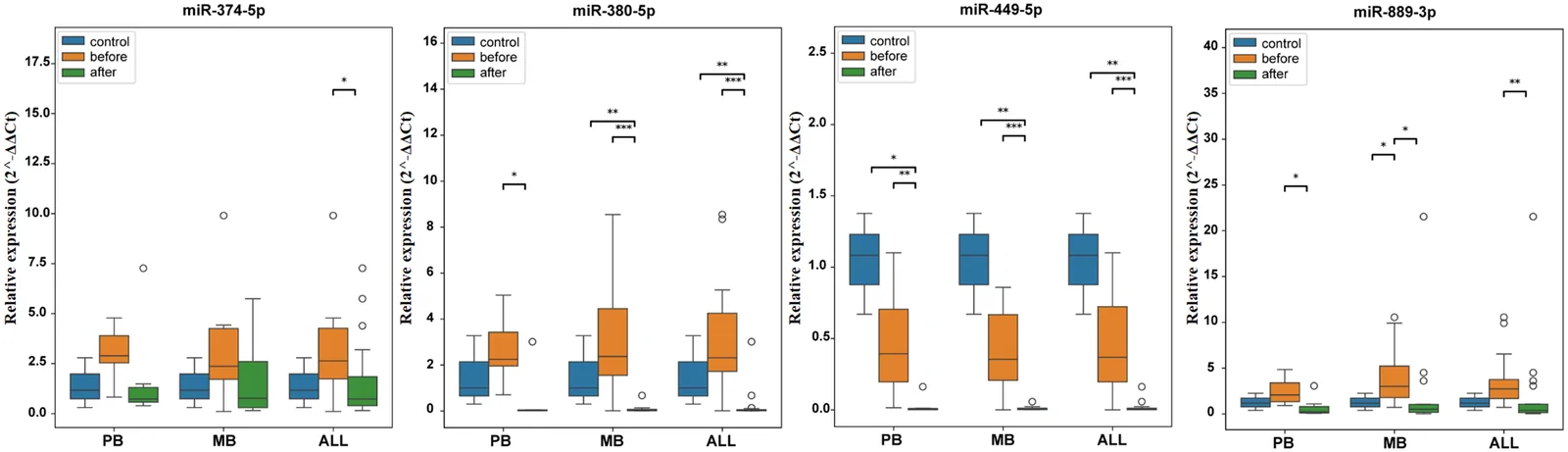
Relative expression of selected miRNAs in leprosy patients before and after multidrug therapy (MDT). Relative expression levels of miR-374-5p, miR-380-5p, miR-449-5p, and miR-889-3p were measured by quantitative real-time PCR (qRT-PCR) in serum samples from healthy controls and patients with leprosy. Expression values were calculated using the 2^-ΔΔCt method with U6 as the internal reference. Patients were categorized into paucibacillary (PB) and multibacillary (MB) groups, and an overall patient group (ALL) representing the combined PB and MB cohorts was also analyzed. Expression levels were compared before and after MDT. Data are presented as mean ± SD. Sample sizes were: healthy controls (n = 3), PB patients (n = 7), and MB patients (n = 14).Statistical significance is indicated as:*P < 0.05, **P < 0.01, ***P < 0.001. PB: paucibacillary; MB: multibacillary.

To validate the microarray findings, the expression levels of miR-374-5p, miR-380-5p, miR-449-5p, and miR-889-3p were examined by qRT-PCR in serum samples from leprosy patients and healthy controls. Among these candidates, miR-889-3p showed the most consistent differential expression pattern. In MB patients, miR-889-3p expression was significantly higher before treatment compared with healthy controls. After completion of multidrug therapy (MDT), miR-889-3p expression significantly decreased, returning to levels comparable to those observed in healthy controls. In contrast, no significant difference in miR-889-3p expression was observed between PB patients and healthy controls. These findings suggest that miR-889-3p expression is associated with disease severity and responds to therapeutic intervention, indicating its potential value as a biomarker for monitoring treatment response (Fig 4 and S2 Table).

### Target validation of SLC35E1

**Fig 5:**
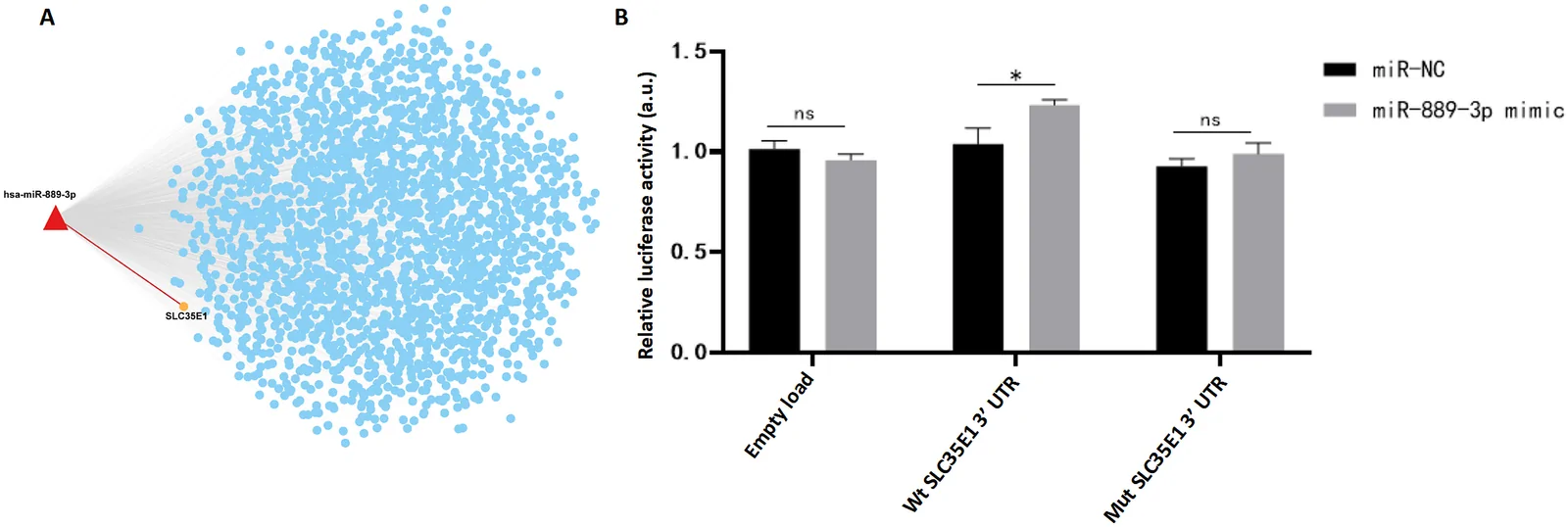
Interaction between miR-889-3p and its predicted target gene SLC35E1. (A) Predicted interaction network of miR-889-3p and its potential target genes based on the top predicted targets identified using the TargetScan database. (B) Dual-luciferase reporter assay showing the interaction between miR-889-3p and the predicted binding site within the SLC35E1 3′UTR. Relative luciferase activity is expressed as the Firefly/Renilla luciferase ratio (arbitrary units, a.u.). miR-889-3p significantly increased reporter activity in the wild-type SLC35E1 3′UTR construct, whereas no significant change was observed in the empty-vector or mutant reporter constructs. These findings support a sequence-specific interaction between miR-889-3p and the SLC35E1 3′UTR.

Bioinformatic prediction using the TargetScan database identified SLC35E1 as a potential target gene of miR-889-3p. Dual-luciferase reporter assays showed that miR-889-3p significantly increased the relative luciferase activity (Firefly/Renilla, arbitrary units [a.u.]) of the wild-type SLC35E1 3′UTR reporter construct, whereas no significant effect was observed in the empty-vector or mutant reporter constructs. These findings indicate that miR-889-3p specifically interacts with the predicted binding site within the SLC35E1 3′UTR, supporting a direct regulatory interaction between miR-889-3p and SLC35E1 (Fig 5).

### Functional enrichment analysis of miR-889-3p target genes

**Fig 6:**
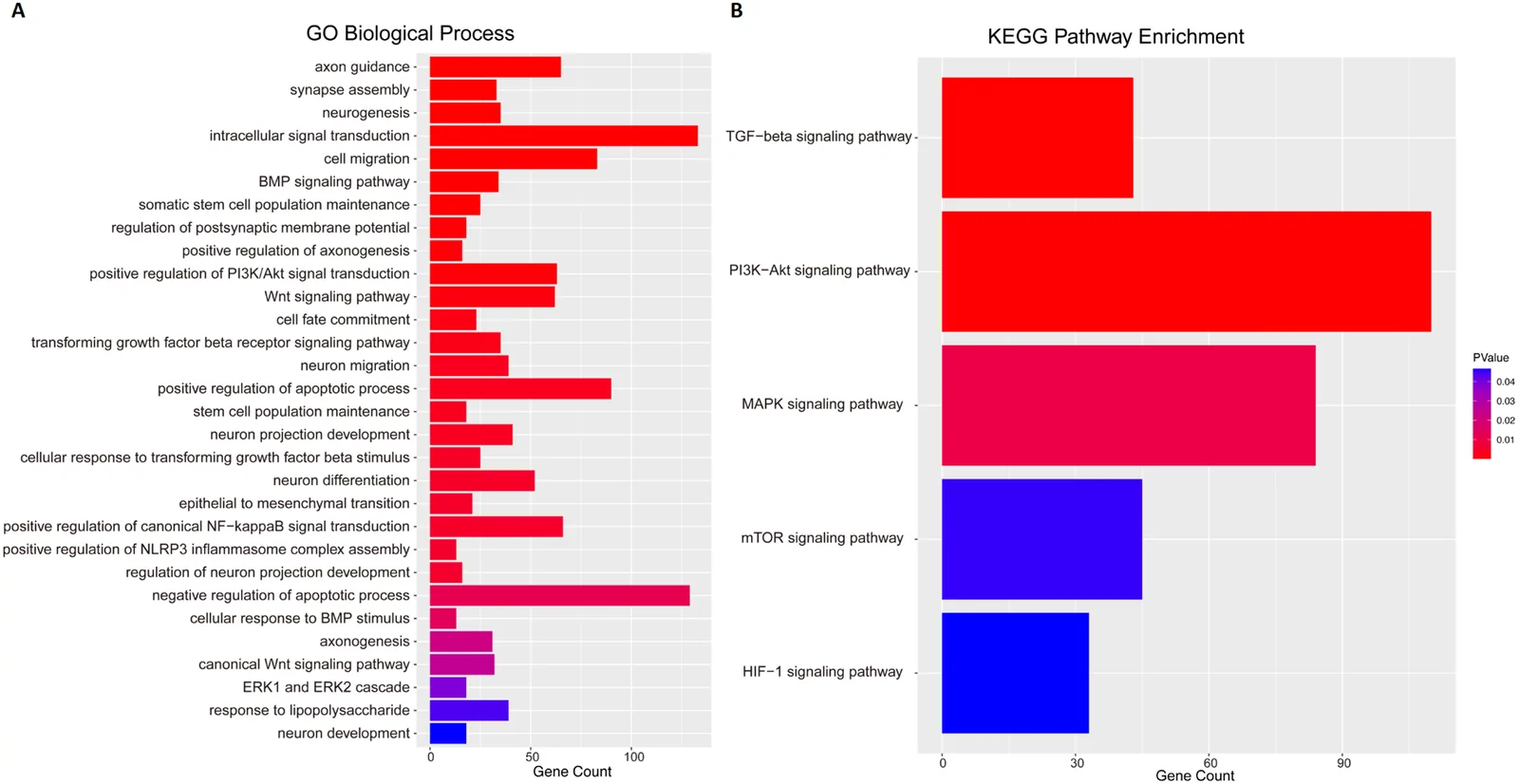
Functional enrichment analysis of predicted miR-889-3p target genes. (A) Gene Ontology (GO) enrichment analysis showing biological processes associated with predicted miR-889-3p target genes. (B) Kyoto Encyclopedia of Genes and Genomes (KEGG) pathway enrichment analysis showing signaling pathways potentially regulated by miR-889-3p target genes. GO: Gene Ontology; KEGG: Kyoto Encyclopedia of Genes and Genomes.

To explore the potential biological functions associated with miR-889-3p, the predicted target genes were subjected to Gene Ontology (GO) and Kyoto Encyclopedia of Genes and Genomes (KEGG) pathway enrichment analysis. GO analysis indicated that these genes were mainly involved in cellular metabolic processes, signal transduction, and immune-related biological processes. KEGG pathway analysis further suggested that miR-889-3p target genes were enriched in several signaling pathways associated with host immune responses and cellular regulation (Fig 6).

### SLC35E1 expression in leprosy skin lesions

**Fig 7:**
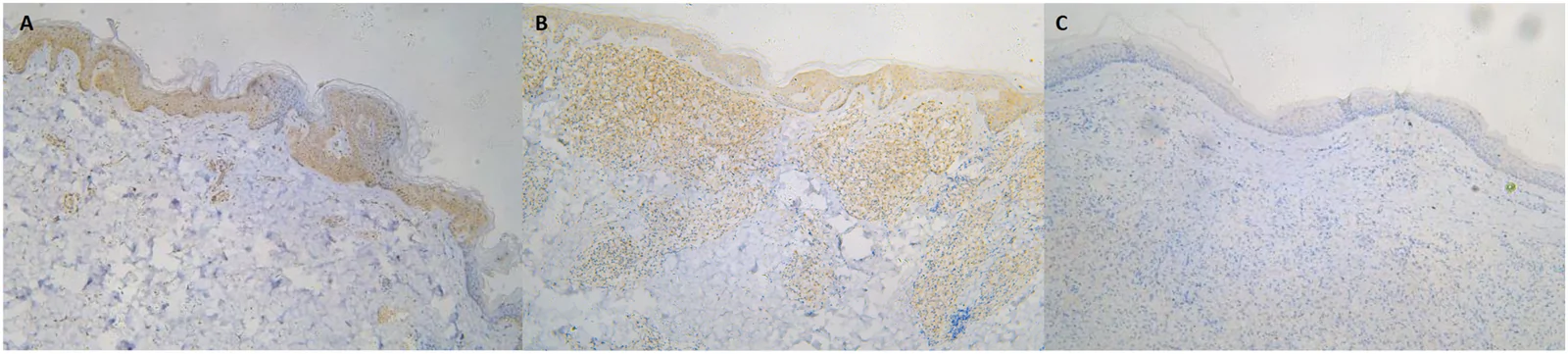
Immunohistochemical detection of SLC35E1 expression in skin lesions of leprosy patients. Representative immunohistochemical staining images showing SLC35E1 expression in normal skin and leprosy lesions. (A) Normal human skin. (B) Skin lesions from leprosy patients before MDT. (C) Skin lesions from leprosy patients after MDT. Brown staining indicates positive SLC35E1 expression. Tissue sections were stained with an anti-SLC35E1 antibody and visualized using a standard immunohistochemical detection method. Images were captured at ×200 magnification.

Immunohistochemical analysis was performed on paired skin biopsy specimens from 16 leprosy patients and 10 normal skin samples. SLC35E1 protein expression was detected in both the epidermis and dermis. Compared with normal skin, leprosy lesions showed significantly increased SLC35E1 expression before treatment. Following MDT, SLC35E1 expression levels decreased in both the epidermis and dermis. Quantitative analysis showed that the mean expression level of SLC35E1 in the epidermis decreased from 6.54 ± 0.74 before treatment to 6.04 ± 0.31 after treatment (P = 0.019). Similarly, dermal expression decreased from 5.14 ± 1.89 to 4.16 ± 1.12 after treatment (P = 0.031) (Fig 7 and S3 Table)

### Correlation between SLC35E1 expression and bacterial burden

**Fig 8:**
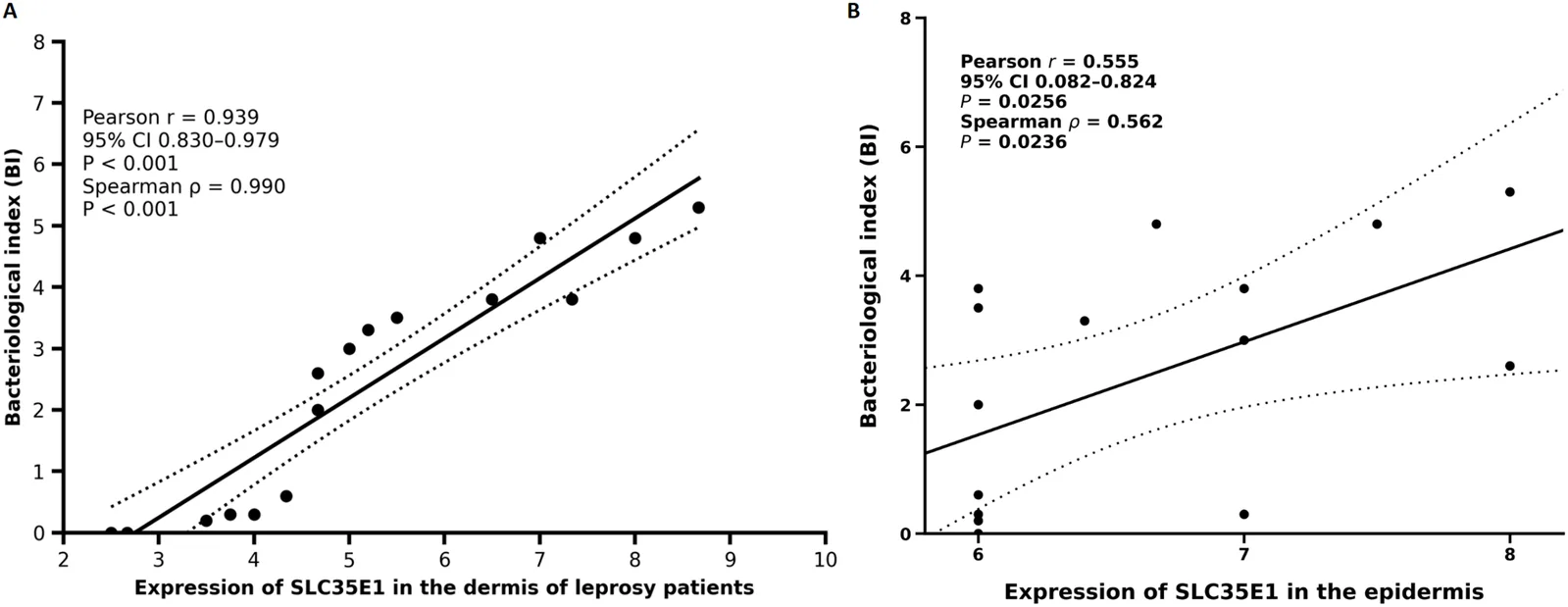
Correlation between SLC35E1 expression and bacterial burden in leprosy lesions. Scatter plots showing the correlation between SLC35E1 expression levels (H-score) and the bacteriological index (BI) in leprosy lesions. SLC35E1 expression exhibited a positive correlation with BI values.Statistical analysis was performed using Pearson correlation analysis. BI: bacteriological index.

Correlation analysis was performed to examine the relationship between SLC35E1 expression and the bacteriological index (BI). SLC35E1 expression in both the epidermis and dermis showed a positive correlation with BI values. In particular, dermal SLC35E1 expression exhibited a strong positive correlation with BI (r = 0.926, P < 0.001), whereas epidermal expression showed a moderate positive correlation (r = 0.584, P = 0.018). These findings suggest that higher SLC35E1 expression is associated with increased bacterial burden in leprosy lesions (Fig 8).

## Discussion

In this study, we systematically profiled miRNA expression across multiple biological compartments, including serum, skin lesions, and nasal mucosa, in patients with leprosy. Among the identified candidates, miR-889-3p emerged as one of the most consistently upregulated miRNAs, particularly in multibacillary (MB) patients. Importantly, its expression decreased significantly following multidrug therapy (MDT), suggesting a potential association with disease activity and treatment-related changes. These observations indicate that miR-889-3p may participate in host responses to Mycobacterium leprae infection and may reflect alterations in disease status during therapy.

Previous studies have increasingly highlighted the role of microRNAs in the immunopathogenesis of leprosy and other infectious diseases. MicroRNAs regulate host immune responses and influence pathogen survival strategies through post-transcriptional control of gene expression [12]. For example, Soares et al. reported a comprehensive microarray analysis of leprosy skin lesions and identified 64 differentially expressed miRNAs, including immune-related miRNAs such as miR-142-3p, miR-146b-5p, miR-342-3p, and miR-361-3p, suggesting their involvement in inflammatory and immune regulatory pathways [18]. Other miRNome analyses using next-generation sequencing have also revealed extensive miRNA dysregulation in leprosy tissues, implicating miRNA-mediated regulation in immune activation, apoptosis, and Schwann cell dysfunction [2]. Furthermore, profiling studies have proposed several miRNAs as potential diagnostic indicators; for instance, a combination of miR-101, miR-196b, miR-27b, and miR-29c has been reported to discriminate between leprosy patients and healthy controls with relatively high sensitivity and specificity [18]. Together, these findings support the concept that miRNA dysregulation represents an important regulatory layer in host responses to M. leprae infection.

Our findings extend previous observations by identifying miR-889-3p as a miRNA associated with bacterial burden and treatment status in leprosy. In addition to expression profiling, we investigated its potential molecular interaction with SLC35E1. Dual-luciferase reporter assays demonstrated that miR-889-3p specifically interacted with the predicted binding site within the SLC35E1 3′ untranslated region (3′UTR), as mutation of this binding site abolished the observed effect, supporting a sequence-specific regulatory interaction between miR-889-3p and SLC35E1. Immunohistochemical analysis further showed that SLC35E1 expression was elevated in leprosy lesions and positively correlated with the bacteriological index (BI), suggesting that SLC35E1 expression is associated with bacterial burden in affected tissues.

SLC35E1 belongs to the solute carrier family and participates in nucleotide sugar transport and cellular homeostasis [19,20]. Recent studies have suggested that SLC35E1 may contribute to skin-related biological processes, including keratinocyte proliferation and inflammatory responses in psoriasis [15]. Although the biological function of SLC35E1 during M. leprae infection remains unclear, these findings raise the possibility that SLC35E1 may participate in host responses associated with inflammatory and tissue-remodeling processes in leprosy.

The relationship between miRNA expression and target gene abundance in clinical tissues is complex and cannot always be predicted solely from canonical miRNA-mediated repression. Infection-induced transcriptional activation, inflammatory signaling, and tissue microenvironmental changes may simultaneously influence target gene expression. Similar context-dependent regulatory patterns have been described in mycobacterial infections. For example, M. leprae-induced miR-21 directly targets components of the vitamin D–dependent antimicrobial pathway and contributes to bacterial persistence, whereas other inflammation-related miRNAs, such as miR-146a, regulate IRAK1/TRAF6 signaling and modulate immune responses in a context-dependent manner [13,14]. These findings indicate that miRNA-mediated regulation during infection reflects the integration of multiple molecular pathways rather than a simple inverse relationship between miRNA abundance and target gene expression.

Consistent with this concept, although miR-889-3p expression was increased in patients with MB leprosy, SLC35E1 expression was also elevated in leprosy lesions and positively correlated with BI. This observation does not necessarily indicate positive regulation of SLC35E1 by miR-889-3p. Instead, the expression pattern observed in clinical tissues likely reflects the combined effects of pathogen-driven immune activation, inflammatory signaling, cellular composition changes, and post-transcriptional regulatory networks. Host gene expression during M. leprae infection is regulated by multiple biological processes, including immune activation and tissue microenvironmental remodeling [1,5]. Therefore, our findings support the existence of a miR-889-3p/SLC35E1 regulatory interaction and suggest that this axis may be involved in host responses during M. leprae infection, while the precise biological consequences require further investigation using disease-relevant cellular and experimental models.

Several methodological considerations and limitations should be acknowledged. Although U6 is widely used as an internal reference gene for miRNA qRT-PCR analyses, its stability was not formally evaluated in the present cohort and may vary across biological sample types [9]. In addition, the microarray analysis was performed as an exploratory screening step with a limited sample size, and retrospective multiple-testing correction could not be applied because the original raw data were unavailable. Therefore, the microarray findings should be regarded as hypothesis-generating and were subsequently validated by qRT-PCR. The relatively small number of healthy controls and the absence of dermatological disease controls may also limit the statistical power and specificity of the findings. Moreover, the dual-luciferase assay performed in HEK293T cells confirmed the interaction between miR-889-3p and the SLC35E1 3′UTR but does not provide direct functional evidence in disease-relevant cell types. Future studies using larger independent cohorts and relevant experimental models are warranted to further clarify the biological significance and clinical relevance of the miR-889-3p/SLC35E1 axis in leprosy.

Taken together, the present study provides preliminary evidence that miR-889-3p is associated with disease status and treatment-related changes in leprosy and may participate in host regulatory responses through interaction with SLC35E1. These findings expand the current understanding of miRNA-mediated regulatory mechanisms in M. leprae infection and highlight the potential relevance of the miR-889-3p/SLC35E1 axis in disease biology.

## Conclusion

Our study suggests that miR-889-3p is upregulated in patients with leprosy, particularly in those with multibacillary disease, and decreases following multidrug therapy (MDT). Dual-luciferase reporter assays support a sequence-specific interaction between miR-889-3p and the SLC35E1 3′UTR, while immunohistochemical analysis demonstrates that SLC35E1 expression in leprosy lesions is associated with the bacteriological index. These findings suggest that miR-889-3p may participate in host responses during Mycobacterium leprae infection and may represent a candidate molecular marker reflecting disease status and treatment-related changes. However, given the exploratory nature of this study, limited sample size, and lack of disease-relevant functional models, the biological mechanisms and clinical utility of the miR-889-3p/SLC35E1 axis require further validation in larger prospective longitudinal cohorts and independent populations.

## Supporting information

S1 Table Primer sequences used for qRT-PCR. Primer sequences used for quantitative real-time PCR validation of miRNAs analyzed in this study.
(XLS)

S2 Table Statistical analysis of qRT-PCR data. Summary of statistical comparisons for miRNA expression among healthy controls, PB, and MB patients before and after MDT.
(XLS)

S3 Table Quantitative analysis of SLC35E1 immunohistochemical staining. H-score values for epidermal and dermal SLC35E1 expression in normal skin and paired leprosy lesions before and after multidrug therapy.
(XLS)
